# Optimizing strength training protocols in young females: A comparative study of velocity-based and percentage-based training programs

**DOI:** 10.1016/j.heliyon.2024.e30644

**Published:** 2024-05-03

**Authors:** Carlo Rossi, Isidora Vasiljevic, Marko Manojlovic, Tatjana Trivic, Marijana Ranisavljev, Valdemar Stajer, Ewan Thomas, Antonino Bianco, Patrik Drid

**Affiliations:** a Sport and Exercise Research Unit, Department of Psychology, Educational Science and Human Movement, University of Palermo, Via Giovanni Pascoli 6, 90144, Palermo, Italy; b Faculty of Sport and Physical Education, University of Novi Sad, 21000 Novi Sad, Serbia

**Keywords:** Muscle damage, Neuromuscular performance, Speed-based training, Traditional exercise, Women, Exercise

## Abstract

The purpose of this study was to compare the effects of velocity-based strength training (VBT) and percentage-based strength training (PBT) on absolute strength, explosive strength, speed, and agility, as well as markers of muscle damage after 6 weeks of exercise programs. The study included 30 young female individuals, divided into three groups of 10 participants: VBT, PBT, and control group. The main findings indicated that the VBT group and PBT group showed significant improvement in 1RM squat exercise (Δ% 27.87 and Δ% 8.98, respectively) and 1RM bench press (Δ% 14.47 and Δ% 8.65, respectively), but a greater enhancement was observed in the VBT group. In addition, VBT induced substantial changes in SJ (Δ% 14.32) and CMJ height (Δ% 7.69), while PBT had an improvement only in the SJ test (Δ% 6.72). The improvement noted in the VBT group could be attributed to its ability to tailor training intensity according to the speed of movement execution. This approach allows athletes to perform each repetition as fast as possible, thus maintaining an optimal intensity for explosive strength development. The capacity of VBT to adapt training intensity based on the speed of movement execution may be the key factor contributing to these results. Therefore, coaches and athletes should consider implementing VBT as a valuable tool to optimize strength and power development. In conclusion, VBT induced greater improvement in the 1RM squat, 1RM bench press, SJ, and CMJ compared to the group that performed the traditional strength training modality. Therefore, VBT is considered a more effective training tool regarding the development of absolute and explosive strength in young women.

## 1 Introduction

Many sports require the ability to generate large force in a relatively short period of time [1]. In particular, muscular strength is one of the most important components involved in the manifestation of power [2]. Moreover, power is considered the ability to generate a great force over a certain distance by rapid muscle contraction [3]. However, in mechanics, mechanical power is calculated as the ratio of work done per unit of time. Of note, available literature also indicates that there is a positive correlation between strength and power [4]. To increase muscle strength, it is important to focus initially on developing maximal strength and then maintaining this level over time. This is critical to achieving significant long-term strength improvements [4,5]. Maximum strength is defined as the highest level of force achieved via muscle contraction and is limited by the relationship between strength and velocity [5]. Strength training is considered as an essential tool for increasing the muscle force. Moreover, for detailed and precise programming of strength training, several variables must be adequately designed, including training volume, intensity, frequency, as well as type of implemented exercise interventions. Manipulation of these variables contributes to a different physiological response related to strength training [6]. It is necessary to emphasize that exercise intensity is one of the most relevant variables concerning improvements in strength parameters [7]. The loads during resistance training programs are most commonly prescribed as a percentage of one repetition maximum (%1RM). Velocity-based training (VBT) is a method that emphasizes the speed at which a weight is lifted or moved to regulate and optimize resistance training [[8], [9], [10]]. It involves measuring the speed of a movement during strength exercises, such as squats or bench press, and using this data to adjust the load of the exercise in real-time. In fact, the main concept of this exercise modality refers to the adjustment of training intensity based on lifting speed, which allows for more precise control over the effort exerted and potentially more personalized training for athletes. Additionally, VBT can be used in various contexts, depending on the athlete's goals and type of training. For example, it can be employed as an adjunct to traditional percentage-based training, providing visual or verbal speed feedback to improve performance and increase motivation and competitiveness. Likewise, VBT can be implemented in all phases of a resistance training program, supporting the prescription of load, sets, number of repetitions and the programming method applied [11]. Further, during sports activities of varying intensity, muscular damage is elicited due to multiple factors, such as mechanical stress, metabolic strain, and the repercussions of prolonged, intense muscular exertion [12]. During muscle damage, the sarcomere, cytoskeletal elements, and sarcolemma are impaired [13]. Muscle damage leads to increased concentrations of enzymes such as creatine kinase (CK) or myoglobin [14,15]. The damage itself affects reduced muscle function in terms of muscle activation and force production. Of note, an increase in total CK value following intense exercise occurs due to damage to skeletal muscle structures at the sarcomere and Z-disc levels [14,16,17]. Comparing the effects of different strength training modalities is considered crucial to understanding which exercise intervention might induce greater benefits in terms of improving neuromuscular performance and reducing muscle damage in physically active female individuals. In addition, this research could help create more targeted and personalized training programs adjusted to the specific needs of young women with a view to optimizing their results in fitness and sports. Hence, the objective of this study was to compare the impact of VBT and percentage-based training (PBT) on absolute strength, explosive strength, speed, and agility, as well as markers of muscle damage in young females following 6 weeks of exercise programs. It was hypothesized that VBT will be more effective concerning the influence on neuromuscular performance compared to PBT. The second hypothesis was that VBT will elicit significantly lower muscle damage relative to the PBT.

## 2 Materials and methods

### 2.1 Study design and participants Characteristics

A total of 30 female individuals, students of the Faculty of Sport and Physical Education of the University of Novi Sad, Serbia, participated in this investigation. The following eligibility criteria have been implemented: (1) the participants needed to have at least 2 times per week of experience in strength training, practicing this modality of exercise 2–3 times per week or more; (2) no injuries of musculoskeletal system were reported before administring of the exercise interventions; (3) illness or health conditions that should impaired examined performance have not been registered; and (4) participants did not use drugs or prohibited substances. Further, the involved population was randomly divided using a random draw with assigned numbers into three groups, two experimental and a third control: the VBT group (Age 22.70 ± 0.82 years; Weight 62.85 ± 5.51 kg); PBT group (Age 22.69 ± 0.69 years; Weight 65.16 ± 8.59 kg); and control group (Age 22.70 ± 0.82 years; Weight 68.21 ± 8.92 kg). Subjects were instructed not to participate in any other strength training programs during the experiment. Additionally, it was not allowed to use supplementation that potentially affected evaluated performances. Before the start of the experimental procedure, participants signed an informed consent for participation in the research. All subjects completed the physical activity readiness questionnaire before the start of the experiment, to identify individuals who should consult a doctor before starting the exercise programs. The study received the ethics committee's approval from the Faculty of Sports and Physical Education of the University of Novi Sad, Serbia (Ref. No. 49-10-02-2023-1) and adhered to the standards of the Declaration of Helsinki.

## 3 Testing procedures

### 3.1 Body weight evaluation

A digital scale was used to measure the body weight of participants (Omron 511BF, OMRON HEALTHCARE, Kyoto, Japan*)*. The subjects were in underwear and barefoot during the measurement. In addition, during the measurement, the subjects stood still, with their weight evenly distributed on both legs and their arms extended at their sides. The result is read with an accuracy of 0.1 kg.

### 3.2 Neuromuscular performance tests

1RM test: For squat and bench press, a 20 kg bar was used (Eleiko; Halmstad, Sweden). Subjects had a warm-up including 5 min on an exercise bike (Wattbike, West Bridgeford, UK: 60 rpm, 60 W) and dynamic stretching with the bar. 1RM was measured with 3–5 min breaks between sets. Subjects executed controlled eccentric contractions followed by maximal force and speed concentric contractions. The average initial load for squat and bench press was 40 kg and 25 kg, respectively. In both tests the load was increased with each set up to 1RM. It was carried out the 5-point 1 RM test, with percentages of 40, 60, 80, 90 % and 1 RM. 1RM squat was performed on the Smith Machine, while 1RM bench press was performed on the bench with free weights. Speed, power and range of motion were assessed in each repetition.

*Squat on the Smith machine:* After standing with knees and hips fully extended, participants perform the controlled squat to a 90° angle between thigh and calf. Then, the participants return to the starting position with maximum concentric contraction. Squat depth is measured for each individual. Lower extremity strength and power are evaluated.

*Bench press:* The subjects lie with their back on a flat bench, feet resting on the floor. Hands are placed on the bar, slightly wider than shoulder width (position adjustments are individual for each subject). The participants were asked to touch the chest with a bar with a controlled eccentric contraction. The participant could not bounce the bar off their chest or lift their shoulders or torso off the bench. After the eccentric contraction, the subjects must push the bar, VBT group as fast as possible, up to the starting position where the elbows are fully extended and the shoulders are in contact with the bench [18]. The absolute resistance is evaluated with the applied test.

*Squat Jump (SJ):* Subjects start in a semi-squat position (knee joint at 90°), hands are on hips, and perform a maximum vertical jump, maintaining extended legs during the jump. This assesses isolated concentric muscle contraction and explosive strength of lower limbs [19]. Three attempts were carried out with a 3-min break; the best score was analyzed. The jump height was the measured parameter. The test was carried out with the Optojump device (Microgate, Mahopac, NY, USA), an optical detection system composed of 2 bars: a transmitting and a receiving one.

*Countermovement Jump (CMJ):* Following a quick half-squat from standing, participants perform a maximal vertical jump. This test assesses the lengthening-shortening cycle, elastic energy transfer, and explosive strength of lower limb extensor muscles [19]. Three attempts were made with a 3-min break; the best score was used. The jump height was the evaluated variable. Also, this test was carried out with the Optojump device (Microgate, Mahopac, NY, USA).

*5- and 20-*m *sprint tests:* These tests measure the speed and explosive strength of the lower limbs. The subject starts 30 cm from the starting line and runs 20 m in the shortest possible time, measured by light gates. Two attempts are made with a maximum break of 2 min.

*Agility test 505:* Participants start 30 cm behind the starting line, run 20 m, change direction, and run another 5 m. Time is measured using light gates. Two trials are performed for right and left directions, with 1 min rest between repetitions.

### 3.3 Assessment of markers of muscle damage

Before the first training episode, a blood sample was taken to determine the initial values of the analyzed parameters of muscle damage, including CK and creatine kinase isoenzyme (CK-MB) (muscle isoenzyme). Fifteen minutes after the end of the first training session, the subjects underwent blood sampling again to establish the acute effects of applied exercise interventions. The third and final blood sample was performed 24 h after the last training episode to determine the chronic effects of the training process on blood markers.

### 3.4 Training procedures

*Warm-up:* Female individuals began with a standardized warm-up of 5 min of cycling (Wattbike, West Bridgeford, United Kingdom) followed by a set of various flexibility and mobility exercises.

**Fig. 1:**
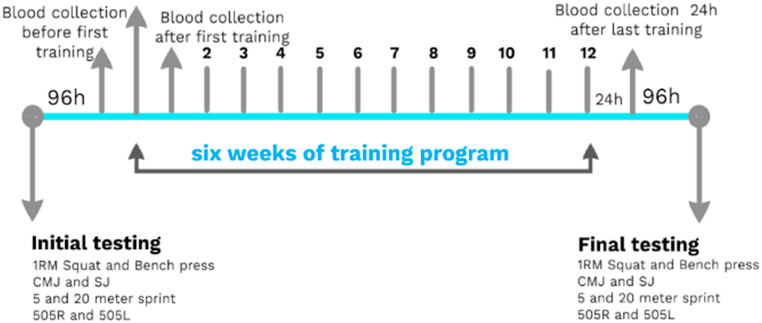
Experimental protocol.

*Testing Sequence:* Baseline testing was conducted 96 h before the experiment. Subjects underwent various neuromuscular tests, including CMJ, SJ, and 505 for both legs, followed by 1RM tests for squat on Smith machine and bench press. Final testing occurred after the last training session and a 96-h break, following the same order as the initial measurement. The dependent variables were T5 and T20 tests. The full experimental procedure is given in Fig. 1.

*Training Groups and Exercise Protocol:* The VBT group used velocity-based strength training adjusting load according to individual load-velocity profile with a 10 % velocity loss limit, the PBT adhered to traditional strength training based on 1RM, while CON did not carry out any type of training. Two experimental groups conducted strength training programs for 6 weeks, two training sessions per week, including a total of 12 exercise episodes. Moreover, a wave periodization structure was applied, modifying relative load (%1RM), number of sets, and rest intervals between groups.

*Training sessions:* The first training episode refers to the first training session of each of the 6 weeks of the experimental procedure and the second training session refers to the second training episode of each of the 6 weeks of the experimental protocol. In the first training session, the following exercises were administered: squat exercise, explosive squat-jump with a bar (20 kg), bench press, plyometric push-ups, and lunges. For the second training session, young female individuals performed the following exercises: squat exercise, explosive squat-jump with a bar (20 kg), bench press, plyometric push-ups, and hip trust exercise that was performed with a bar (20 kg), and additional load that represented the body mass of each participants. The order of exercises was the same during the 6 weeks of the experimental procedure. The full description of all exercises and training programs is available in the supplementary material.

*Load Velocity Profile (LVP) formation:* During the 1RM assessment, execution velocities during each repetition were recorded. For sets that included multiple repetitions, the fastest repetition was used to determine LVP. Lunge velocity while performing 1RM was not included in the analysis to determine LVP. Individual LVP is predicted to be formed based on the average speed achieved with loads between 20 and 90 % of 1RM [20]. The LVP was formed (using Microsoft Excel 2016, Microsoft, Redmond, Washington, USA) by entering the relative load and average concentric speed achieved during that repetition into the table, to form the line that best fits the data based on the linear regression equation. Further, a table was created showing the load in relation to %1RM and the speed corresponding to that load. Individual LVP parameters were used in the training process for VBT groups.

*Blood sampling and analysis:* Blood samples were collected from the anterior cubital vein. Five ml venous blood samples with gel and clot activator were collected in tubes (BD vacutainer) for determination of CK and CK-MB activity. As previously emphasized, muscle damage markers were assessed across three independent time points (Fig. 1).

### 3.5 Statistical analysis

A priori sample size calculation was performed using G-power (G*Power version 3.1.9.4, effect size = 0.3, 1 − β = 0.80, α = 0.05), which demonstrated that a minimum of 30 participants was necessary to determine training effects and differences between groups. Data analysis was conducted using SPSS 20 (Statistical Package for Social Science, Chicago, IL, USA). The statistical level of significance was set at p ≤ 0.05. Values of all variables are expressed as mean ± standard deviation (SD). The Shapiro-Wilk test was used to verify the normality of the data distribution. One-way ANOVA and Independent-Samples T-test examined baseline differences between groups regarding neuromuscular performances and markers of muscle damage, respectively. Two-way repeated-measures ANOVA with Bonferroni post-hoc correction was employed to determine between-groups differences in these parameters. Paired-Sample T-test explored the differences within each group for all analyzed variables. Partial eta squared (η^2^ₚ) was computed as a measure of effect size and interpreted as small, moderate, and large, for values η^2^ₚ ∼ 0.01, η^2^ₚ ∼0.06, and η^2^ₚ ≥ 0.14, respectively [21]. Delta percentage (Δ%) was calculated using the following formula: Δ% = final-baseline/baseline x 100. The graphs are designed with the Microsoft Office Excel package (version 2019, Microsoft Inc. Italy).

## 4 Results

**Fig. 2:**
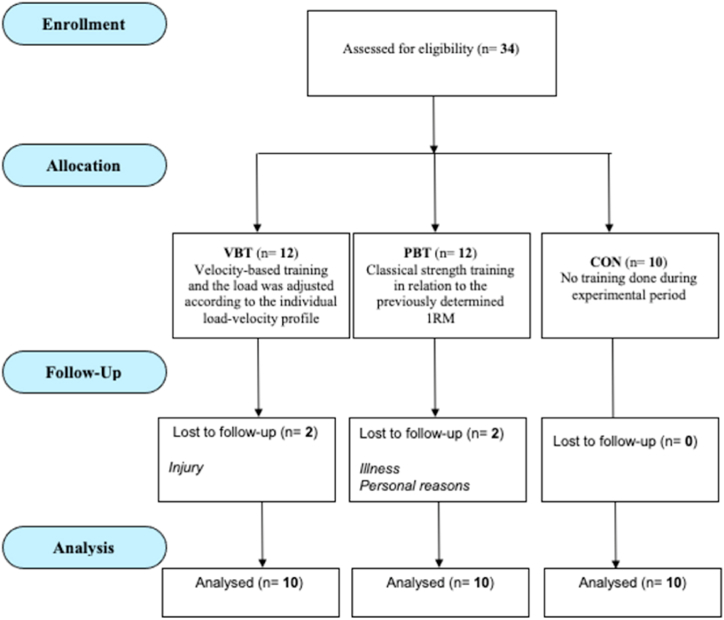
Flow chart of the participant's selection process.

Initially, 34 female physically active students were assessed for eligibility. As presented in Fig. 2, four females did not complete the experimental procedure. Specifically, two participants withdrew due to an injury experienced outside of the study, and two females were lost to follow-up due to illness and personal reasons. Overall, 10 participants in each of the groups finished 6 weeks of training programs and were included in this research.

No between-group differences were recorded at the baseline testing regarding all neuromuscular variables and markers of muscle damage. Although a significant group × time interaction was found for the variables 1RM squat (F = 31.521; η^2^ₚ = 0.700; p < 0.001), 1RM bench press (F = 3.370; η^2^ₚ = 0.250; p = 0.049) and SJ (F = 5.323; η^2^ₚ = 0.283; p = 0.011), there were no differences between analyzed groups. Additionally, no statistically significant differences between groups were revealed for the CMJ test, 5-m and 20-m speed tests, and 505L and 505D tests (Table 1).

**Table 1:** Pre and post of the parameters analyzed in the three groups of the study.

Outcome variables	VBT (=10)			PBT (=10)			CON (=10)			**ANOVA**		
	Pre	Post	Δ%	Pre	Post	Δ%	Pre	Post	Δ%	G	T	G x T
Squat *(kg)*	66 ± 12.50	84.40 ± 11.90*	27.87	66.80 ± 7.91	72.80 ± 9.02*	8.98	65 ± 9.74	65.10 ± 8.31	0.15	0.083	0.000	0.000
**Bench Press***(kg)*	38.70 ± 8.68	44.30 ± 6.66*	**14.47**	39.30 ± 7.27	42.70 ± 8.75^#^	**8.65**	37.20 ± 7.14	38.30 ± 5.49	2.95	0.457	0**.000**	0**.049**
**SJ***(cm)*	24.78 ± 5.40	28.33 ± 5.13*	**14.32**	25.28 ± 3.06	26.98 ± 2.27^§^	**6.72**	25.45 ± 2.42	26.03 ± 1.91	2.27	0.873	0**.000**	0**.011**
**CMJ***(cm)*	25.98 ± 3.03	27.98 ± 2.74^#^	**7.69**	26.52 ± 3.86	27.39 ± 2.91	3.28	25.93 ± 2.40	26.68 ± 2.14	2.89	0.827	0**.001**	0.265
**T5m***(s)*	1.16 ± 0.05	1.14 ± 0.06	−1.72	1.17 ± 0.07	1.15 ± 0.05	−1.70	1.17 ± 0.08	1.17 ± 0.07	0	0.785	0.391	0.724
**T20m***(s)*	3.55 ± 0.14	3.52 ± 0.14	−0.84	3.60 ± 0.11	3.57 ± 0.12	−0.83	3.63 ± 0.19	3.63 ± 0.14	0	0.311	0.324	0.853
**505R***(s)*	2.85 ± 0.15	2.80 ± 0.07	−1.75	2.91 ± 0.16	2.86 ± 0.16	−1.71	2.87 ± 0.17	2.71 ± 0.59	−5.57	0.526	0.249	0.760
**505L***(s)*	2.82 ± 0.16	2.79 ± 0.12	−1,06	2.91 ± 0.15	2.89 ± 0.15	−0.68	2.91 ± 0.12	2.94 ± 0.11	1.03	0.142	0.521	0.292

Legend: VBT - Velocity-based training group; PBT - Percentage-based training group; CON – control group.

a - statistically significant difference between first and second measurement (p-value <0.001)**.**

b - statistically significant difference between first and second measurement (p-value <0.01)**.**

c - statistically significant difference between first and second measurement (p-value <0.05); G - group effect; T - time effect; G x T - group–time interaction; Δ% - delta percentage; SJ – Squat jump; CMJ – Countermovement jump; T5m – 5-m sprint tests; T20 m - 20-m sprint tests; 505R - Agility test 505 Right; 505L - Agility test 505 Left.

Specifically, although differences between baseline and final measurements were observed within VBT and PBT groups for 1RM squat, 1RM bench press, and SJ, greater improvement was noted in the VBT group (1RM squat; Δ% 27.87 and Δ% 8.98, respectively; 1RM bench press; Δ% 14.47 and Δ% 8.65, respectively, and SJ; Δ% 14.32 and Δ% 6.72, respectively. Only the VBT group demonstrated changes regarding the CMJ test (Δ% 7.69). No statistically significant differences within experimental groups were found for speed and agility tests. In the control group, there were no differences between initial and final testing in all variables (Table 1).

**Fig. 3:**
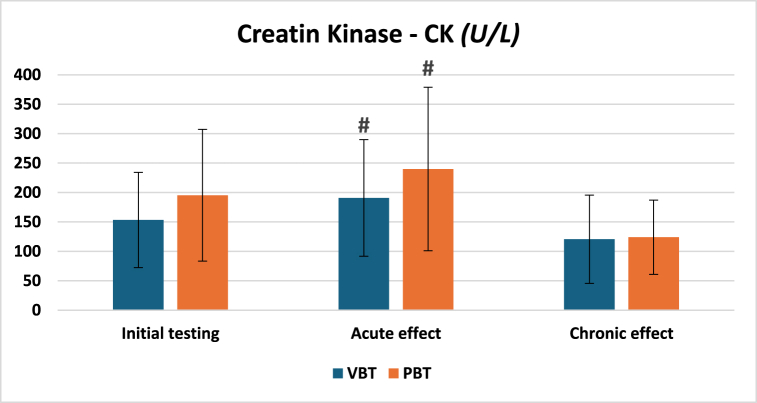
Values of creatine kinase across three-time points.-VBT – Velocity-based training group; PBT - Percentage-based training group; U/L - micro/Liter; # - A statistically significant difference between first and second measurement (p-value <0.01).

**Fig. 4:**
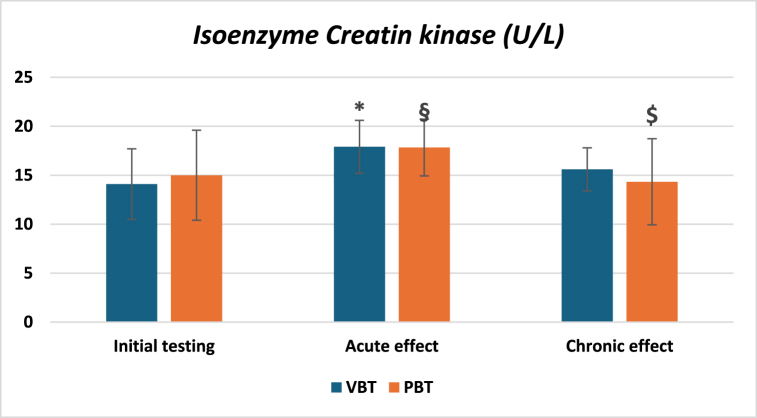
Values of CK-MB isoenzymes across three-time points.-VBT – Velocity-based training group; PBT - Percentage-based training group; U/L - micro/Liter; $ - A statistically significant difference between second and third measurement (p-value <0.05) for PBT group; § - A statistically significant difference between first and second measurement (p-value <0.05) for PBT group; * - A statistically significant difference between first and second measurement for VBT group (p-value <0.001).

Regarding the markers of muscle damage, no between groups differences were revealed for both markers across each phase. However, statistically significant differences were observed in CK between the first and second measurements for the VBT (153.30 U/L and 190.80 U/L, respectively) and PBT (195.33 U/L and 240 U/L, respectively) groups (Fig. 3). Furthermore, in Fig. 4, concerning the CK-MB, differences were recorded between the first and the second measurement (14.10 U/L and 17.90 U/L, respectively) in the VBT group, while in the PBT group, statistically significant differences were obtained between the first and the second measurement (15 U/L and 17.83 U/L, respectively), as well as between the second and the third (17.83 U/L and 14.33 U/L, respectively). Additionally, it should be highlighted that a tendency toward statistically significant differences in CK-MB was found between chronic and acute effects for the VBT group (17.9 U/L and 15.6 U/L, respectively, p = 0.084).

## 5 Discussion

The goal of this investigation was to determine the effects and differences between VBT and PBT regarding absolute strength, explosive strength, speed, and agility after six weeks of experimental programs in young female individuals. Partially consistent with the first hypothesis, although no differences were observed between the groups in terms of all neuromuscular tests, VBT produced greater improvement in absolute and explosive strength than PBT. More specifically, VBT was more effective in relation to improvements in 1RM squat, 1RM bench press, and SJ test than PBT. Furthermore, only VBT positively altered values in the CMJ test of young women. In all groups, there were no differences between baseline and final measurements in tests of speed and agility. On the other hand, contrary to the second hypothesis, the implemented exercise interventions induced similar muscle damage at each analyzed phase. Indeed, numerous studies compared the efficiency of VBT and PBT programs concerning various neuromuscular performances [[22], [23], [24], [25], [26], [27], [28]]. More precisely, available scientific evidence is partially consistent with the findings of the presented study. For instance, a meta-analysis conducted by Liao et al. [23] did not reveal statistically significant differences between VBT and PBT regarding effects on absolute strength, explosive strength, and speed. Moreover, no statistically significant differences in 1RM squat were observed between VBT and traditional strength training modality in the sport-collegiate female basketball players [25]. In addition, in line with the results obtained in this investigation, speed-based strength training elicited greater improvements in jump performances of female athletes, including CMJ and SJ tests relative to the PBT program. On the other hand, several studies reported significant differences between VBT and PBT interventions relating to effects on neuromuscular performances [27,28]. Specifically, a statistically significant group × time interaction between two strength training modalities was found for the 1RM bench press and CMJ test in favor of the VBT group in trained male individuals [27]. Likewise, speed-based strength training intervention was more efficient compared to the PBT program in terms of enhancement of absolute strength in highly trained rowers [28]. The discrepancy between the findings of the present study and available literature can be potentially explained by examined population. Namely, this research involved physically active female students, while the mentioned studies were conducted on highly trained male athletes. In summary, as already highlighted, VBT induced greater improvements in 1RM squat, 1RM bench press, and explosive strength relative to the traditional strength training program. In addition, the implemented strength training interventions had similar effects on speed and agility. The specific implementation of VBT during the concentric phase of the movement appears to have driven more substantial adaptations, elucidating the greater enhancement in absolute and explosive strength compared to the traditional training method. Overall, future studies are warranted to more clearly understand mechanisms responsible for more favorable adaptations in absolute strength and explosiveness in the VBT group relative to PBT in young female individuals. Although there were no differences between groups, values of CK and CK-MB markers changed significantly at certain phases. In terms of acute training effects, a significant increase in both markers of muscle damage was observed between the first and second measurements for the VBT group. These results are consistent with the available literature [[29], [30], [31]]. For instance, Chapman et al. [31] reported that a substantial increase in CK levels was observed 24 h following the implementation of the VBT program in recreationally active males. On the other hand, in the PBT group, in addition to the recorded acute training effects for both analyzed markers, a significant decrease in CK-MB levels was revealed at the third measurement. Additionally, a tendency towards statistically significant differences between acute and chronic training effects for CK-MB was noted in the VBT group (p = 0.084). Most importantly, the results concerning biochemical markers demonstrated that the participants in both exercise groups had a similar metabolic response to the training load. Moreover, taking into account highlighted findings particularly related to the chronic training effects, it appears that applied exercise interventions did not induce significant damage to the muscle tissue. Overall, in the future, it is essential to improve knowledge regarding alterations of markers of muscle damage following different strength training modalities in young female individuals. Lastly, it is necessary to emphasize that this is one of the first studies that included an analysis of markers of muscle damage after the implementation of VBT in the female population. Regarding the practical implications of the findings, it is indispensable to highlight that the results obtained are very useful for exercise specialists engaged in sports that require a high level of absolute or explosive strength. Specifically, due to greater improvements in tests that evaluated mentioned neuromuscular performances, female athletes should more commonly implement VBT than PBT program. Overall, in the future, strength and conditioning coaches should create training programs emphasizing the application of VBT regarding improvements in absolute and explosive strength performance. Lastly, in terms of muscle damage, considering that VBT and PBT elicited similar acute and chronic training effects, strength and conditioning coaches can apply strength training irrespective of its modality.

In conclusion, it is necessary to emphasize that although no differences between groups were found concerning all implemented neuromuscular tests, VBT induced greater enhancement in 1RM squat, 1RM bench press, SJ, and CMJ compared to the group that performed traditional strength training modality. Therefore, VBT is considered a more effective training tool regarding the development of absolute and explosive strength in young females. For this reason, strength and conditioning specialists need to take into account the highlighted facts and implement VBT to achieve substantial improvements in the mentioned neuromuscular performances. Finally, regarding markers of muscle damage, considering that there were no differences between analyzed groups across all time points, a more comprehensive analysis of muscle damage is warranted in the future to verify these findings.

**STRENGTHS AND LIMITATIONS OF THE STUDY** The present study has strengths that are indispensable to emphasize. This was one of the first investigations that compared the effects of VBT and PBT programs on neuromuscular performances in exclusively female individuals. Moreover, the study provided a comprehensive analysis of neuromuscular performances, considering that eight tests evaluated absolute and explosive strength, as well as speed and agility. Concerning methodological aspects, it should be noted that the study had enough statistical power to detect exercise effects and that a control group of young females was also involved. Contrary, some limitations should be acknowledged. The current study was conducted on a sample of physically active young female students of sports sciences. Thus, the following investigations should address elite female athletes. In addition, only two markers evaluated muscle damage in the analyzed population. Based on the highlighted restriction, it is apparent that more studies are needed to verify findings related to muscle damage.

### 5.1 Research support

This research received no external financial or non-financial support.

### 5.2 Relationships

There are no additional relationships to disclose.

### 5.3 Patents and Intellectual Property

There are no patents to disclose.

### 5.4 Other activities

There are no additional activities to disclose.

## Supplementary materials

The supplementary document about Training sessions is available.

## Data availability statement

Data will be made available on request by the corresponding author.

## CRediT authorship contribution statement

**Carlo Rossi:** Writing – original draft, Methodology, Investigation, Conceptualization. **Isidora Vasiljevic:** Writing – original draft, Investigation, Conceptualization. **Marko Manojlovic:** Methodology. **Tatjana Trivic:** Methodology. **Marijana Ranisavljev:** Writing – review & editing, Investigation. **Valdemar Stajer:** Supervision. **Ewan Thomas:** Writing – review & editing. **Antonino Bianco:** Supervision. **Patrik Drid:** Supervision.

## Declaration of competing interest

The authors declare that they have no known competing financial interests or personal relationships that could have appeared to influence the work reported in this paper.
