# Supplementary material for: Optimizing strength training protocols in young females: A comparative study of velocity-based and percentage-based training programs
Source: Heliyon. 2024 May 3;10(9):e30644. doi: 10.1016/j.heliyon.2024.e30644 (PMC11089354; doi:10.1016/j.heliyon.2024.e30644)
Supplement: Multimedia component 1 [file mmc1.docx]

Training process

|  | Week 1 | | Week 2 | | Week 3 | | Week 4 | | Week 5 | | Week 6 | |
| --- | --- | --- | --- | --- | --- | --- | --- | --- | --- | --- | --- | --- |
| Exercise | **Reps** | **% 1RM** | **Reps** | **% 1RM** | **Reps** | **% 1RM** | **Reps** | **% 1RM** | **Reps** | **% 1RM** | **Reps** | **% 1RM** |
| Training episode 1 |  |  |  |  |  |  |  |  |  |  |  |  |
| Squat | 8, 8, 8 | 70, 70, 70 | 8, 6, 5 | 70, 75, 80 | 6, 5, 3 | 75, 83, 88 | 8, 6, 5 | 70, 75, 80 | 6, 5, 3 | 78, 85, 90 | 5, 3, 2+ | 85, 90, 95 |
| Bench press | 8, 8, 8 | 70, 70, 70 | 8, 6, 5 | 70, 75, 80 | 6, 5, 3 | 75, 83, 88 | 8, 6, 5 | 70, 75, 80 | 6, 5, 3 | 78, 85, 90 | 5, 3, 2+ | 85, 90, 95 |
| Squat-jump (with bar) | 2 (3), 2 (3) | EB | 2 (3), 2 (3) | EB | 2 (3), 2 (3) | EB | 2 (3), 2 (3) | EB | 2 (3), 2 (3) | EB | 2 (3), 2 (3) | EB |
| Plio push-ups | 2 (3), 2 (3) | BM | 2 (3), 2 (3) | BM | 2 (3), 2 (3) | BM | 2 (3), 2 (3) | BM | 2 (3), 2 (3) | BM | 2 (3), 2 (3) | BM |
| Lunge forward | 10, 10, 10 |  | 10, 10, 10 |  | 10, 10, 10 |  | 10, 10, 10 |  | 10, 10, 10 |  | 10, 10, 10 |  |
| Training episode 2 |  |  |  |  |  |  |  |  |  |  |  |  |
| Squat | 8, 8, 8 | 70, 70, 70 | 8, 6, 5 | 70, 75, 80 | 6, 5, 3+ | 75, 83, 88 | 8, 6, 5 | 70, 75, 82 | 6, 4, 2 | 78, 88, 92 | 4, 4, 4 | 70, 70, 70 |
| Bench press | 8, 8, 8 | 70, 70, 70 | 8, 6, 5 | 70, 75, 80 | 6, 5, 3+ | 75, 83, 88 | 8, 6, 5 | 70, 75, 82 | 6, 4, 2 | 78, 88, 92 | 4, 4, 4 | 70, 70, 70 |
| Squat-jump (with bar) | 2 (3), 2 (3) | EB | 2 (3), 2 (3) | EB | 2 (3), 2 (3) | EB | 2 (3), 2 (3) | EM | 2 (3), 2 (3) | EM |  |  |
| Plio push-ups | 2 (3), 2 (3) | BM | 2 (3), 2 (3) | BM | 2 (3), 2 (3) | BM | 2 (3), 2 (3) | BM | 2 (3), 2 (3) | BM |  |  |
| Hip trust | 8, 8, 8 | +BM | 8, 8, 8 | +BM | 8, 8, 8 | +BM | 8, 8, 8 | +BM | 8, 8, 8 | +BM |  |  |

***Legend****: %1RM (% from 1 repetition maximum), BM – body mass 2 (3) = cluster set, 2 x 3 reps, +BM – + own body mass on the bar, EB – empty bar – 20 kg*

**Training sessions:** First, the squat exercise was performed, the execution technique of which was previously described in the manuscript, and then the explosive squat-jump exercise with a bar (20 kg) was conducted. The subjects performed three maximal vertical jumps from a squat with a barbell on the shoulders and then took a 15-second break, followed again by 3 maximal jumps. The break between two sets of this exercise lasted 2 minutes. The participants determined the depth of their squat based on their individual preferences, and the resulting jump visually resembled the CMJ. After the jumps, bench presses were implemented (the execution technique was explained in the neuromuscular performance tests section), three sets with a two-minute rest between sets. The fourth exercise was plyometric push-ups. The participant had to perform three push-ups, in which, following the concentric phase of the movement which involved pushing off the surface, followed by the flight phase and the return to push-ups. The respondents performed three maximum repetitions, and then took a 15-second break, to perform three maximum repetitions again. The break between the two sets was 2 minutes. The last exercise of the first training session of the week was a lunge. The lunge was performed with an additional load distributed equally on both arms, calculated based on the individual maximum repetition according to the following formula: [21] ((6RM)*(0.52)+14, 82kg)*0.6. While performing a lunge, the participants were required that the knee of the landing leg not touch the ground but be relatively close to it; in that position, the angle of the upper leg and lower leg of both knees should form a 90° angle. The subjects alternately performed repetitions with the right and left leg. In addition to the described exercise program, instead of the lunges, the second training session included the hip thrust exercise. This exercise was performed with a bar (20 kg), and additional load that represented the body mass of each respondent. All subjects' starting position was seated, with the scapular region resting on the bench and the legs bent at the knees, while the bar was positioned in the hip flexor area. The respondents pushed the bar upwards with a hip extension movement and then returned it to the starting position with a controlled eccentric contraction. The rest between sets lasted 2 minutes and between repetitions 2 seconds.
